# Aerosols in the Pre-industrial Atmosphere

**DOI:** 10.1007/s40641-017-0061-2

**Published:** 2017-03-11

**Authors:** Kenneth S. Carslaw, Hamish Gordon, Douglas S. Hamilton, Jill S. Johnson, Leighton A. Regayre, M. Yoshioka, Kirsty J. Pringle

**Affiliations:** 1 School of Earth and Environment, University of Leeds, Leeds, UK; 2 College of Agriculture and Life Sciences, Cornell University, Ithaca, New York USA

**Keywords:** Aerosol, Climate, Pollution, Biosphere, Radiative forcing, Climate sensitivity

## Abstract

**Purpose of Review:**

We assess the current understanding of the state and behaviour of aerosols under pre-industrial conditions and the importance for climate.

**Recent Findings:**

Studies show that the magnitude of anthropogenic aerosol radiative forcing over the industrial period calculated by climate models is strongly affected by the abundance and properties of aerosols in the pre-industrial atmosphere. The low concentration of aerosol particles under relatively pristine conditions means that global mean cloud albedo may have been twice as sensitive to changes in natural aerosol emissions under pre-industrial conditions compared to present-day conditions. Consequently, the discovery of new aerosol formation processes and revisions to aerosol emissions have large effects on simulated historical aerosol radiative forcing.

**Summary:**

We review what is known about the microphysical, chemical, and radiative properties of aerosols in the pre-industrial atmosphere and the processes that control them. Aerosol properties were controlled by a combination of natural emissions, modification of the natural emissions by human activities such as land-use change, and anthropogenic emissions from biofuel combustion and early industrial processes. Although aerosol concentrations were lower in the pre-industrial atmosphere than today, model simulations show that relatively high aerosol concentrations could have been maintained over continental regions due to biogenically controlled new particle formation and wildfires. Despite the importance of pre-industrial aerosols for historical climate change, the relevant processes and emissions are given relatively little consideration in climate models, and there have been very few attempts to evaluate them. Consequently, we have very low confidence in the ability of models to simulate the aerosol conditions that form the baseline for historical climate simulations. Nevertheless, it is clear that the 1850s should be regarded as an early industrial reference period, and the aerosol forcing calculated from this period is smaller than the forcing since 1750. Improvements in historical reconstructions of natural and early anthropogenic emissions, exploitation of new Earth system models, and a deeper understanding and evaluation of the controlling processes are key aspects to reducing uncertainties in future.

## Introduction

The radiative energy balance of the planet is sensitive to the amount, size, and chemical properties of atmospheric aerosol particles from natural [1, 2] and anthropogenic sources. Changes in anthropogenic emissions over the industrial period have significantly altered the abundance and properties of aerosols and caused a change in radiative energy balance, or radiative forcing, which is estimated to lie between near 0 and −2 W m^−2^ [3]. This large uncertainty in forcing significantly limits our understanding of historical climate change and the reliability with which we can make climate change projections [4, 5].

The abundance, properties, and distribution of aerosols in the pre-industrial (PI) atmosphere are important for climate for two reasons. Firstly, the PI is the reference period used in climate models for calculating the radiative forcing caused by anthropogenic activities, and uncertainty in the aerosol reference state substantially affects the magnitude of the calculated forcing [6, 7]. Secondly, it has been suggested that the global mean climate sensitivity may depend on the sea-surface temperature pattern [8–11], which to a large degree will be controlled by the very uncertain distribution of natural aerosols.

There is insufficient observational evidence to accurately define the state of atmospheric aerosols in the PI, so we mostly rely on estimates from global climate model simulations. With such a lack of observational constraint on models, it is important for simulations to be based on reliable information about aerosol and precursor gas emissions, as well as a comprehensive understanding of aerosol chemical and physical processes in the natural atmosphere.

The uncertainty in model simulations of PI aerosols may not make a large contribution to the calculated forcing uncertainty associated with aerosol-radiation interactions [3, 12] because the magnitude of the forcing depends approximately linearly on the aerosol load [13] (so the perturbation calculated by the model is not strongly dependent on the reference state). However, the radiative forcing caused by aerosol-induced changes in cloud albedo depends on fractional changes in cloud droplet number concentrations according to

$$ \frac{\varDelta A}{A}\approx \frac{\varDelta N}{N}\frac{\left(1- A\right)}{3 A} $$

where *A* is cloud albedo and *N* is droplet number concentration [14, 15]. The consequence of this dependence is that aerosol-cloud forcing over the industrial period is particularly sensitive to cloud droplet concentrations (and hence aerosol concentrations) under PI conditions when concentrations were low. The impact of this high sensitivity has been demonstrated in global models [6, 7, 16], showing up as a large sensitivity of anthropogenic radiative forcing to the emissions of natural aerosols and precursors. The high sensitivity also means that variations in PI climate, normally attributed to volcanic and solar effects [17], will also be affected by variability in tropospheric aerosols. Although the above equation represents only one potential effect of aerosols on cloud microphysics and structure [18], studies show that other radiatively important cloud properties such as cloud top height, liquid water content, and cloud fraction also depend non-linearly on aerosol concentrations [19], with the steepest changes in these properties often occurring under the low-aerosol conditions that typified the PI.

The high sensitivity of forcing to droplet and aerosol concentrations in the PI may explain why some climate models prescribe a minimum droplet concentration. This practice has a large effect on the calculated forcing [20] and will probably have a large bearing on the climate sensitivity of a model that is tuned to reproduce historical temperatures [21]. The practice of tuning models in this way shows that it is important to develop a fundamental understanding of PI aerosols so that we can build models based on a sound physical understanding.

Most interest currently focuses on the effect of aerosols on atmospheric radiation and warm clouds, but there are significant open questions about how ice-nucleating particles may have changed over the industrial period. Ice-nucleating particles are predominantly natural dusts, sea spray, and biological particles [22, 23], although anthropogenic material may contribute [24]. In general, ice-nucleating particle concentrations depend most strongly on the concentrations of large (>0.5 μm) particles [25], which have changed less than smaller more numerous particles over the industrial period [26]. Our understanding of global ice-nucleating particles in terms of particular aerosol components is only just emerging, so we do not attempt to review the PI state of such particles here.

It is important to define what is meant by “pre-industrial” and how it relates to other commonly used reference periods in climate science [27]. The Industrial Revolution started in the UK around the 1780s [28], and the mid-1700s was a period of major changes in agriculture, industry, and population, which led to steep rises in pollutant emissions, albeit with large regional variations. However, the mid-1700s are not a reference for *pre-human* atmospheric conditions [29] because global population was already around 800 million, so land use will already have been modified by human activity [30], which will have affected natural emissions from vegetation and introduced aerosol pollution from biofuel combustion [31]. In fact, ice core records of air pollution predate the Industrial Revolution by centuries [32]. The 1850s are commonly used as the starting point for climate model simulations, probably because it marks the start of the instrumental temperature record [33]. However, by 1850, aerosol emissions were locally already significantly above 1750 levels, and 1850 is normally considered to be the start of the *second* Industrial Revolution. Oddly, 1750 has been used as the reference for climate model calculations of radiative forcing in the context of IPCC [3], but 1850 is used as the reference for starting model simulations. The *Anthropocene* is another definition of global environmental change [34], but the current definition is not very relevant to aerosol pollution and radiative forcing.

The definition of pre-industrial affects the PI to present-day aerosol radiative forcing that is calculated. In one study, the aerosol-cloud radiative forcing was estimated to be −1.42 W m^−2^ with a 1750 reference and −1.30 W m^−2^ with an 1850 reference [6]. It was also shown that about 46% of the aerosol-cloud forcing uncertainty could be attributed to anthropogenic emissions with an 1850 reference but only 34% with a 1750 reference, showing that the small anthropogenic emissions in 1850 contribute to the uncertainty in the calculated forcing. The differences in emissions between 1750 and 1850 are likely to be an underestimate because they neglect many of the additional factors described in later sections of this review.

Very few studies have focused on simulating and evaluating the aerosol properties in the PI period. Climate models simulate PI aerosols as part of their historical simulations, usually using a common set of emissions for either 1750 [31] or in the period 1850–1870 [35]. However, even with common emissions, differences between the models result in a very large range of simulated PI aerosol states. This range is important because it affects the multi-model range of simulated aerosol-cloud forcings over the industrial period by 15–60% [7]. An estimate of the PI aerosol state is also required in studies that use satellite observations to estimate anthropogenic radiative forcing [36]. This approach relies on using observations of aerosol optical depth under present-day clean atmospheric conditions or making assumptions about how natural aerosols contribute to aerosol optical depth at different wavelengths, although the extrapolation back to PI conditions may be unreliable [37]. Furthermore, given the large spatio-temporal heterogeneity in PI aerosol abundance [38], it is not appropriate to define a single PI aerosol reference.

In this review, we describe recent developments in our understanding of aerosols in the PI atmosphere. Although stratospheric aerosols and perturbations to them are an important aspect of the planetary energy balance in the PI [17, 39], we focus on aerosols in the troposphere because of the rapid changes in our understanding of their properties. We summarise key developments in our understanding of the physical and chemical processes of relevance to natural PI-like environments as well as the remaining open questions. There is a lack of dedicated studies of PI aerosols from which the aerosol properties can be defined. We therefore include in our review our best assessment of global PI aerosol properties based on our own model simulations, which also includes an analysis of over 20 sources of uncertainty related to emissions as well as microphysical and chemical processes.

## Measurements of Pre-industrial Aerosols

There are two ways to estimate the state of aerosols in the PI from measurements—either from analysis of aerosol chemical components in ice cores and sediments or by attempting to deduce the properties based on observations of the unpolluted present-day atmosphere. In this section, we briefly review these approaches and what they tell us about PI aerosols.

Some limited information about the abundance and seasonality of aerosols in the PI can be obtained from ice cores. The short atmospheric lifetime of aerosols means that ice core record changes in concentrations that are representative of small regions [40] or perhaps hemispheric scales if the aerosols were pervasive, such as in the Industrial Revolution [41–44]. Commonly analysed ice core aerosol components include black carbon, dust, sulphate, and salt ions (e.g. Ca, K, Na, Mg, and Cl). Some source identification is feasible by analysing other chemical species, such as levoglucosan as a tracer for biomass burning [45], methanesulfonic acid (MSA) for marine biogenic dimethyl sulphide emissions [46, 47], and electric conductivity measurements of acidity for volcanic activity [48, 49].

It is more difficult to estimate atmospheric aerosol concentrations from ice core records than non-reactive greenhouse gas concentrations, such as carbon dioxide, which can be measured directly as the gas mixing ratio inside trapped bubbles [50]. An estimate of PI atmospheric aerosol mass concentrations requires a lot more information, including the local water deposition rate. Furthermore, aerosol transport to remote locations is episodic and controlled by poorly understood chemical transformation and removal processes [51]. These factors make it difficult to relate aerosol concentrations in ice to those that existed in the PI atmosphere, especially since regional meteorology may have been different in the PI [40]. An additional fundamental limitation of ice core records is that they do not record several properties of aerosols most relevant to climate (notably number concentrations of cloud condensation nuclei and particle size distributions). Measurements of the size of insoluble aerosol particles using electron microscopy [52, 53] is a possibility for future studies of PI aerosols, but studies using these techniques are still rare in the literature.

Ice core records from Europe, Greenland, and Antarctica show that sea-salt deposition has remained fairly constant over the industrial period [40, 54, 55]. In contrast, MSA was regionally up to a factor two higher in the PI [40, 56, 57], which has been attributed to changes in Arctic sea-ice cover, greater biological productivity in the colder PI, and possibly changes in gas phase chemistry [40]. Such trends are not accounted for in emission inventories used in global models [31], but model simulations suggest that increases of dimethyl sulphide emissions by 50–100% would have a substantial effect on regional aerosols, clouds, and radiative forcing [6, 58, 59]. Ammonium concentrations increased in the industrial period but with very different temporal changes, and there are multi-decadal variations in the PI that are most likely driven by natural processes [40]. Ice cores show higher black carbon aerosol concentrations from fires in the seventeenth to nineteenth centuries, peaking around the mid-nineteenth century [60, 61], a pattern also seen in the charcoal record [62, 63].

Very few modelling studies have attempted to reconstruct PI aerosols from ice core records. Most comparisons focus on longer timescales, such as those within the Paleoclimate Modelling Intercomparison Project [64]. Atmospheric dust concentrations have received the most attention [65, 66], with studies suggesting large changes in atmospheric dust in response to vegetation changes. Atmospheric black carbon concentrations have been compared with those inferred from a sediment core and deposited black carbon in snow at the D4 Greenland ice core site [67] and Hamilton et al. [68] compared northern hemisphere ice core black carbon concentrations with those from fire modelling simulations, suggesting that atmospheric concentrations of black carbon in the PI are strongly dependent on the assumptions made about fire emissions.

**Fig. 1:**
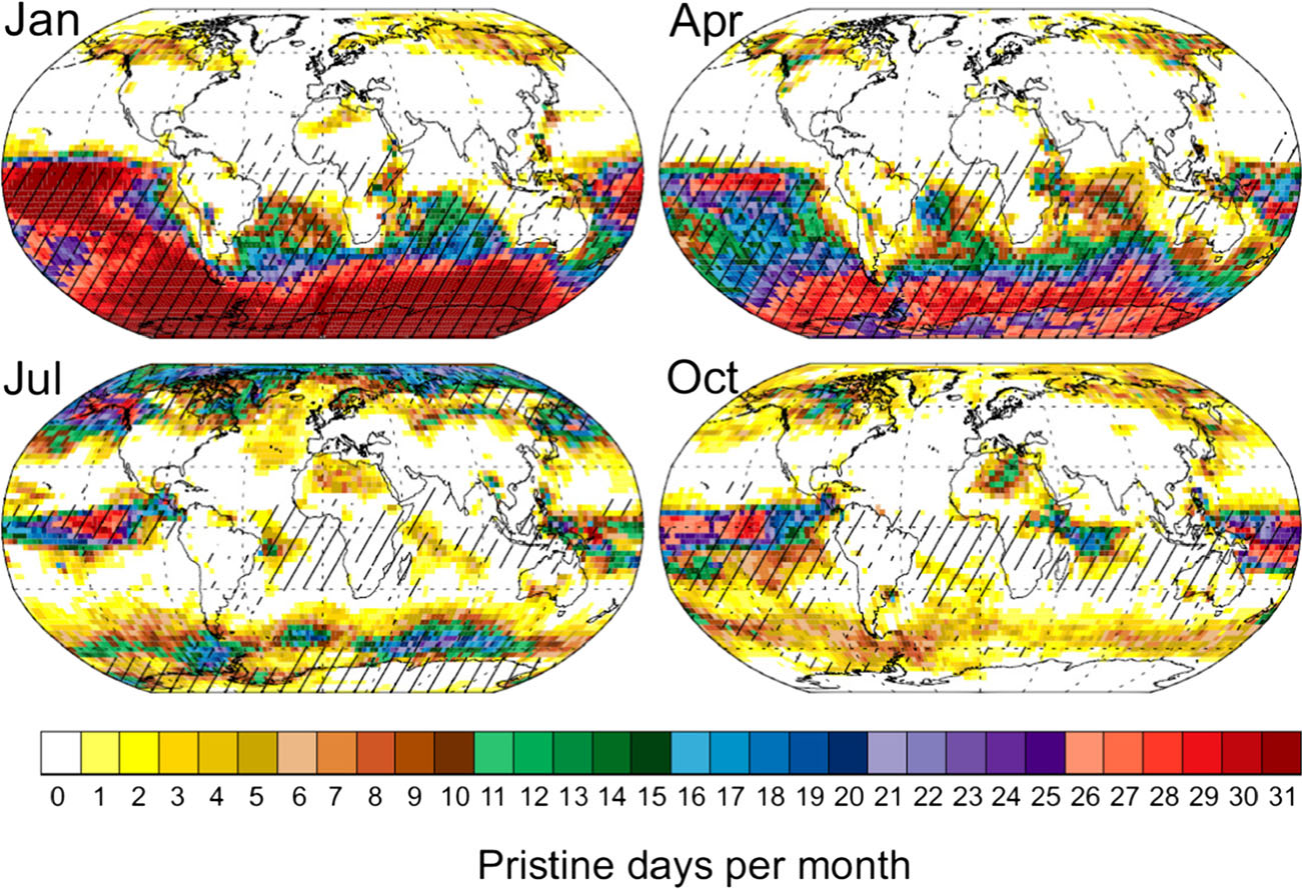
Identification of regions in which cloud condensation nucleus (CCN) concentrations at cloud base (∼915 hPa) are similar in the present day and in the PI. *Colours* show the number of days per month on which PI and present-day CCN concentrations differ by no more than ±20%. The *stippling* shows regions where the sensitivities of PI and present-day CCN to 28 model parameters are similar (*r* ^2^ ≥ 0.9) in that grid cell. From Hamilton et al. [38]

An alternative approach is to estimate PI aerosol properties using a model to identify present-day atmospheric conditions that resemble the PI. In terms of cloud condensation nuclei (CCN) concentrations, it is estimated that up to 12% of today’s Earth’s surface could be representative of the PI [38], with greater occurrences in single months (see Fig. 1). The occurrence of PI-like conditions for other aerosol properties is likely to be different. Most of the PI-like locations for CCN are marine and located in the southern hemisphere, but such regions also occur in some boreal regions. Currently, there is limited overlap of the identified pristine regions with the availability of aerosol measurements [69], especially in terrestrial environments. However, an analysis of baseline aerosol measurement stations in the Global Atmosphere Watch network [26] shows that aerosols over the remote islands of American Samoa and Amsterdam Island may still resemble PI conditions, which could provide opportunities to make PI-like aerosol and precursor gas measurements from established research facilities.

Clean background conditions are often identified in ambient measurements by filtering them to remove signatures of air pollution. Tracers of pollution include black carbon [70], carbon monoxide [71], and aerosol number concentrations [72, 73]. However, a wide range of threshold values is used: for example, black carbon concentrations in the range of 14.2 to 70 ng m^−3^ have been applied [70, 74–77]. Although such approaches can detect “clean air”, none of these tracers is unique to air pollution (e.g. black carbon from natural fires) and it is incorrect to associate the cleanest air with natural conditions. In agreement with our previous study [38], we show below that some PI regions may have had quite high aerosol concentrations. Therefore, it remains unclear how PI aerosol conditions can be detected just using measurements.

## Aerosol Emissions in the Pre-industrial

Aerosol emissions in the PI would have been influenced by three factors: (i) natural emissions and natural variability in these emissions; (ii) anthropogenic modification of natural emissions compared to present-day conditions caused by factors like climate change and modification of natural land cover by anthropogenic land-use change; and (iii) anthropogenic emissions, whether from pre-industrial domestic and agricultural practices (for a 1750 reference) or also including early industrial processes (for an 1850 reference).

Natural aerosol emissions, processes, and their coupling to the Earth system were reviewed in Carslaw et al. [2]. In that review, the potential effect of changes in emissions on 2100 climate was estimated, although we now know that natural emissions in the PI are also important for understanding historical climate [6]. Natural emissions include wind-blown sea spray, soil and desert dust, smoke particles from wildland fires, biogenic organic compounds that are oxidised to form secondary organic aerosol, and sulphate aerosol from various sulphur compounds emitted by volcanic activity and marine phytoplankton. All of these natural emissions show natural variability on a wide range of timescales as well as strong coupling to biogeochemical cycles [78], so we cannot assume they were the same in the PI as now, or the same in 1750 as in 1850. There is also large uncertainty in the emissions, which have been shown to significantly affect the PI aerosol concentrations and radiative forcing [16].

Human modification of natural land cover over the industrial period is known to affect aerosols, trace gases, and climate [79], but the magnitude of these changes already in the period 1750 to 1850 is not well understood. Current estimates of PI land-cover fractions [80, 81] range from very little human land use outside of Western Europe and Southeast Asia in the HYDE 3.1 land-use dataset [30] to extensive human land use across Eurasia, India, Southeast Asia, Central and Northeast America, and Africa in the KK10 dataset [82]. This uncertainty makes it difficult to define truly natural emissions. For example, recent fire modelling incorporating different land-cover scenarios estimates that CCN number concentrations could be a factor 1.6–2.7 times higher in the PI than previously thought [68], with important consequences for anthropogenic aerosol radiative forcing.

Anthropogenic emissions in the PI cannot be neglected. Biofuel combustion for cooking and heat was a large source of PI aerosol pollution in Asia and Europe, and by 1850, the emissions could have been as much as 41% of present-day levels [83]. Furthermore, up to half of the global black carbon burden could be from biofuel emissions from 1850 to 1890 [84]. Earlier, dirtier fuels also released more pollutants to the atmosphere than later more refined fuels, altering emission factors (grams pollutant per kilogram fuel burnt) over time [85].

## Processes Controlling Pre-industrial Aerosols

The amount of aerosol in the PI atmosphere is clearly determined by the emissions. However, it is now realised that the behaviour of the PI aerosol system is likely to have been different to today in many regions. Therefore, we cannot predict climate-relevant aerosol properties in the PI based solely on relative emissions or chemical concentrations in ice cores. Global Earth system models with detailed treatments of aerosol microphysics and chemistry can help to define the aerosol properties [86–90].

A clear demonstration of how the aerosol system has changed is the study of Spracklen and Rap [91] who showed that the existence of anthropogenic aerosols in the northern hemisphere has halved the sensitivity of cloud albedo to changes in natural aerosol emissions. So, in the PI atmosphere, aerosols, clouds, and planetary radiation balance would have been much more sensitive to fluctuations in natural emissions than today.

Approximately half of today’s CCN originate from new particle formation via gas-to-particle conversion or “nucleation”, and up to 80% in some regions [92]. Nucleation is therefore a key process to include in Earth system models that aim to simulate PI aerosols. Rates of nucleation and subsequent condensational growth to CCN size will have been different in the PI because they depend strongly on the concentrations of trace gases such as sulphuric acid and ammonia [93] as well as the surface area of existing aerosols, which scavenge condensing vapours and nuclei [94]. The fraction of nucleated particles that grow to CCN size will have been higher in some places (due to lower probability of loss) but lower in others (due to lower abundance of condensable vapours). At present, the relative effects of these factors are not completely understood even for the present-day atmosphere.

Based on our current understanding of nucleation, we can begin to build a picture of how PI aerosol processes differed from the present day. We know that nucleation is caused by extremely low volatility vapours such as sulphuric acid and highly oxidised organic compounds [93, 95–97], bases like amines and ammonia [93, 98, 99], and ions [93, 100]. The main factor controlling nucleation will be large changes in sulphuric acid vapour concentrations in polluted regions. Since anthropogenic sulphuric acid emissions are roughly constant over the year while biogenic emissions (of both organics and sulphur) are strongly peaked in summer, we speculate that PI aerosol likely had a stronger seasonal cycle in number concentration than the present day [97]. There is not yet enough information to determine how other trace gases and ions may have shaped PI aerosols. The second major factor is the lower sink of nucleating vapours in the PI. Gordon et al. [101] showed that this can allow nucleation of biogenic vapours [100] to increase substantially in the PI, providing a “nucleation buffering” mechanism that raises PI aerosol concentrations above the level that might be expected based on the generally lower emissions. A third factor is that the volatility of biogenic nucleating vapours depends on which species were involved in the oxidation steps (ozone, OH, HO_2_, and NO_3_) as well as the concentrations of NO_*x*_ (ref [102]), which have been strongly affected by anthropogenic activities [103, 104]. This will have affected not just nucleation but also all aerosol chemistry and trends [105].

Changes in the properties of aerosols cannot be considered separately from the changes they induce in clouds. Higher aerosol concentrations lead to smaller cloud droplets and a possible local- to regional-scale suppression of precipitation formation, which is the major loss process of aerosols [106, 107]. There is potential for a feedback in which enhanced aerosol removal in the clean PI further suppresses aerosol concentrations [108, 109], although many other factors need to be considered in regional cloud systems [18]. None of these processes has been explored in any depth in connection with the PI aerosol state.

## What Did Pre-industrial Aerosols Look Like?

Global models provide the only way to estimate the microphysical properties of PI aerosols that are relevant to climate, although more could be done to evaluate some aspects of the models against measurements described earlier.

To provide some idea what global aerosols looked like and how uncertain they are, we have analysed a large set of model simulations of the HadGEMvn8.4 climate model [110] using 1850 emissions (so, according to the discussion above, the reference for many climate model simulations, but with a small amount of pollution that was not present in 1750). The simulations were run for a single year of 2008 meteorology so do not account for any poorly understood changes in meteorology since 1850. Natural emissions are the same as used in previous published studies [6, 31, 111]. The small anthropogenic emissions for 1850 were taken from the Atmospheric Chemistry and Climate Model Intercomparison Project [35]. Following the statistical approach described in previous studies [111, 112], an ensemble of 235 simulations was generated to sample 26 uncertainties in all the aerosol emissions and most of the processes. Then, for each output of interest in each model grid box, a statistical emulator model was constructed to define how the output varies with respect to the 26-dimensional parameter uncertainty space, allowing a full histogram of the output uncertainty to be obtained given the parametric uncertainty of the model.

**Fig. 2:**
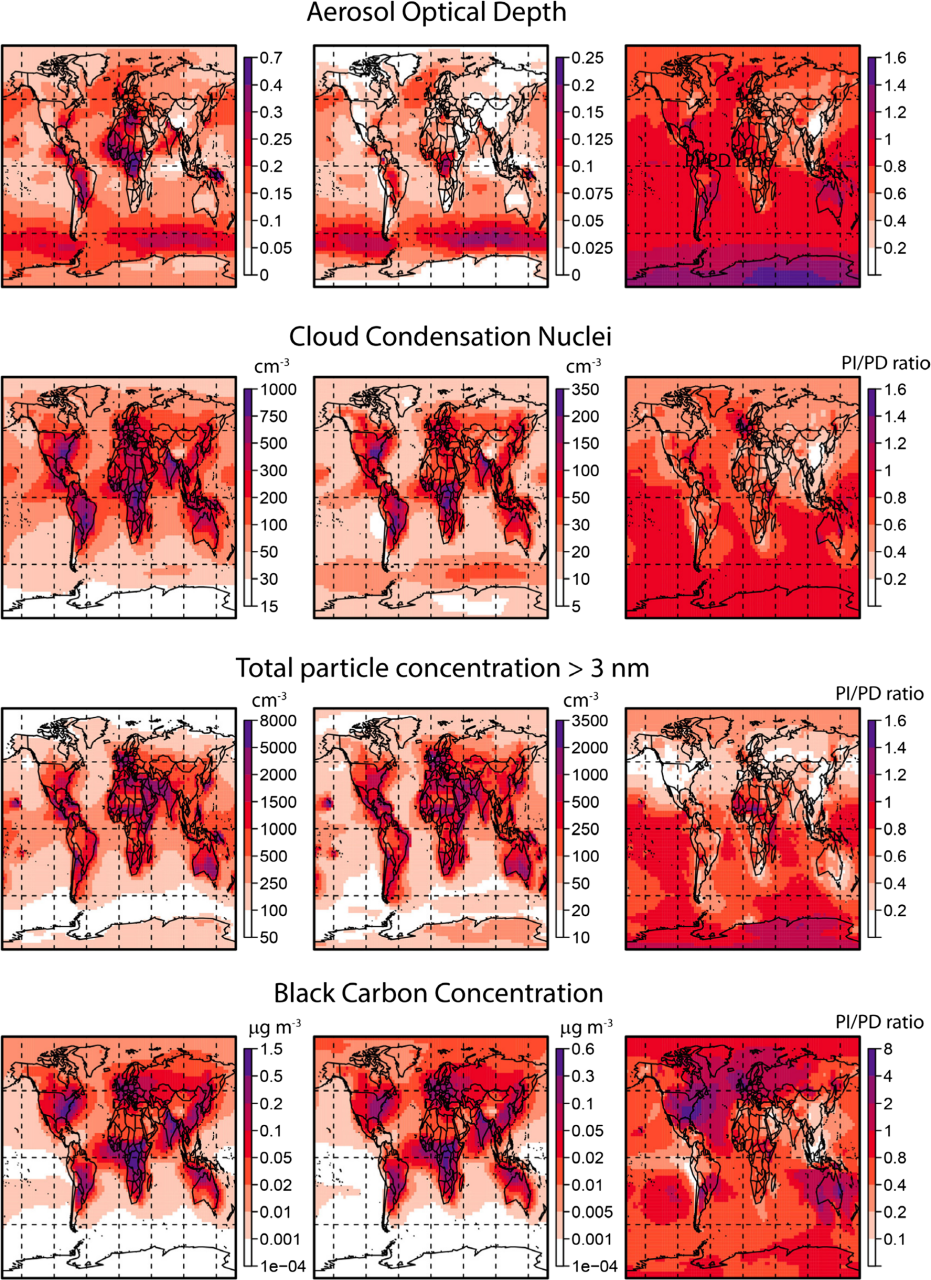
Model calculations of annual mean pre-industrial (1850) aerosol properties: aerosol optical depth (550 nm), cloud condensation nucleus number concentrations at approximate cloud-base altitude of 890 hPa, total particle concentration for particles larger than 3 nm diameter at cloud base, and black carbon mass concentrations at cloud base. For each variable, the *panel on the left* shows the mean value and the *central panel* shows the standard deviation, given the parametric uncertainty in the model. The *panel on the right* shows the ratio of the mean pre-industrial value to the mean present-day value

**Fig. 3:**
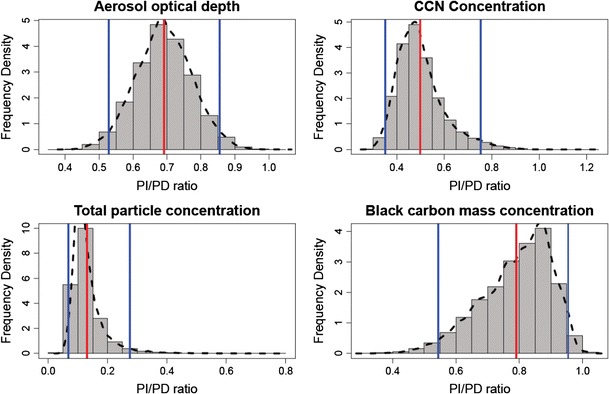
Uncertainty in the ratio of PI to present-day (PD) aerosol properties in boreal Canada (see map in Fig. 4) caused by the parametric uncertainty in the aerosol model. Each plot shows a histogram of the ratio PI/PD. Concentrations are for approximate cloud-base altitude of 890 hPa. The *red vertical line* is the mean of the distribution and the *blue lines* are the 95% confidence intervals

**Fig. 4:**
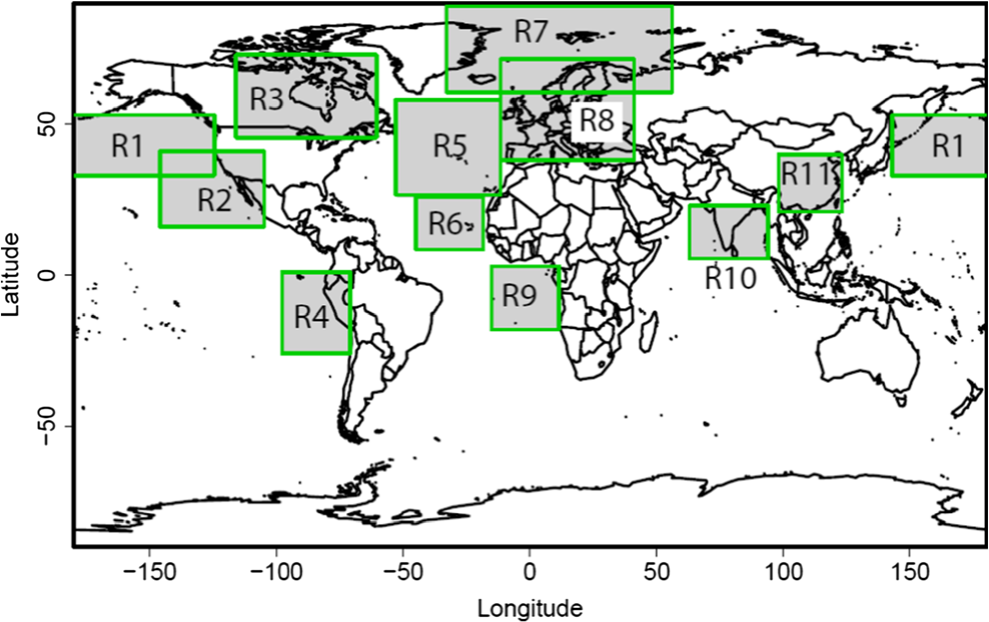
Map of regions analysed in Table 1 and Fig. 7

Figure 2 shows the global distribution of aerosol optical depth and several in situ aerosol properties in the boundary layer: CCN number concentrations, total particle number concentrations (particles larger than 3 nm diameter), and black carbon mass concentrations. We also show the uncertainty in these quantities (as the standard deviation) as well as the ratio to present-day conditions. These ratios are themselves uncertain because of the uncertainty in 1850 and present-day aerosols, so we show examples of the ratio uncertainty in Fig. 3. Table 1 summarises average aerosol properties over selected marine and land regions, which are shown in Fig. 4.

**Table 1:** Modelled aerosol properties in the PI and present day (PD): aerosol optical depth (AOD), CCN concentration at 0.2% supersaturation, concentration of particles larger than 3 nm diameter (N_3_), and black carbon mass concentrations. The values are annual averages over the 11 regions in Fig. 4. The three columns give the mean, the standard deviation from the uncertainty in the model (*σ*), and the ratio of the PI to present-day (PI/PD)

		AOD			CCN_0.2%_/cm^−3^			N_3_/cm^−3^			Black carbon/μg m^−3^		
	Region	Mean	*σ*	PI/PD	Mean	*σ*	PI/PD	Mean	*σ*	PI/PD	Mean	*σ*	PI/PD
R1	N Pacific Ocean (O)	0.16	0.06	0.73	71	18	0.55	184	45	0.22	0.01	0.01	0.50
R2	Pacific off California (O)	0.09	0.03	0.84	108	21	0.57	339	97	0.28	0.02	0.01	0.65
R3	E Canada (L)	0.10	0.03	0.64	122	32	0.53	250	92	0.26	0.06	0.03	1.35
R4	Pacific off S America (O)	0.09	0.03	0.94	93	17	0.65	373	99	0.75	0.004	0.002	0.29
R5	N Atlantic (O)	0.15	0.06	0.83	92	19	0.67	225	65	0.29	0.02	0.01	1.58
R6	N Atlantic off W Africa (O)	0.18	0.06	0.89	130	22	0.73	348	79	0.57	0.03	0.01	1.00
R7	Arctic Ocean (O)	0.14	0.05	0.78	88	23	0.60	192	74	0.38	0.03	0.02	1.25
R8	Europe	0.17	0.05	0.68	252	60	0.68	1116	547	0.33	0.15	0.08	1.45
R9	Atlantic off SW Africa (O)	0.14	0.05	0.76	170	34	0.72	352	65	0.65	0.09	0.05	0.53
R10	Indian Ocean (O)	0.17	0.05	0.53	368	74	0.41	1085	324	0.41	0.20	0.08	0.34
R11	China (L)	0.10	0.03	0.24	243	57	0.21	775	337	0.15	0.15	0.08	0.17

One result of our simulations and earlier studies [38] is that PI aerosol concentrations over terrestrial regions were probably higher than those over the ocean. Some of the land/ocean contrast over Europe and eastern N America is due to early industrial emissions. However, comparison with Fig. 1 of ref. [38], which used 1750 emissions, shows that much of the elevated aerosol concentration over land is due to natural terrestrial aerosol sources [38], which will include wildfires and biogenically driven new particle formation in these simulations [97]. Our simulations therefore suggest that aerosol concentrations would not have been the same over land and ocean as has been assumed for pre-human environments [29]. And certainly by 1850, it is clear that early industrial activity over Europe and eastern North America will have significantly raised aerosol concentrations.

Over northern hemisphere ocean regions, CCN concentrations in 1850 were in the range 50–100 cm^−3^, with an uncertainty of 10–30 cm^−3^. These are about 50–70% of present-day concentrations. Over continental land regions, CCN concentrations peak at about 900 cm^−3^ in regions affected by fires and early industrial pollution, but most continental regions have concentrations in the range 100–300 cm^−3^ except in high-latitude boreal regions where they are lower. Asia stands out as a region in which PI CCN concentrations were considerably lower than today—typically 20–40% of present-day concentrations.

Figure 3 shows how uncertain the ratio of PI to present-day CCN concentrations is at a central N American location. The mean PI to present-day CCN ratio is about 0.5, but the lower and upper 95% confidence intervals lie at 0.35 and 0.75. This means that the uncertainty in the aerosol model has a very substantial effect on our ability to determine how much CCN concentrations have changed over the industrial period.

Black carbon mass concentrations are predicted to be much more similar in the PI and present day than is the case for CCN. Over much of the northern hemisphere, black carbon concentrations in the PI are estimated to be about 80% of present-day values but can exceed present-day values in locations with high early-industrial emissions. Black carbon concentrations also generally have higher uncertainty (a standard deviation about 50% of the mean), which reflects the large uncertainty in emissions. The ratio of PI to present-day concentrations is also more uncertain; for example, Fig. 3 shows that the 95% confidence intervals of the ratio lie at 0.55 and 0.95.

**Fig. 5:**
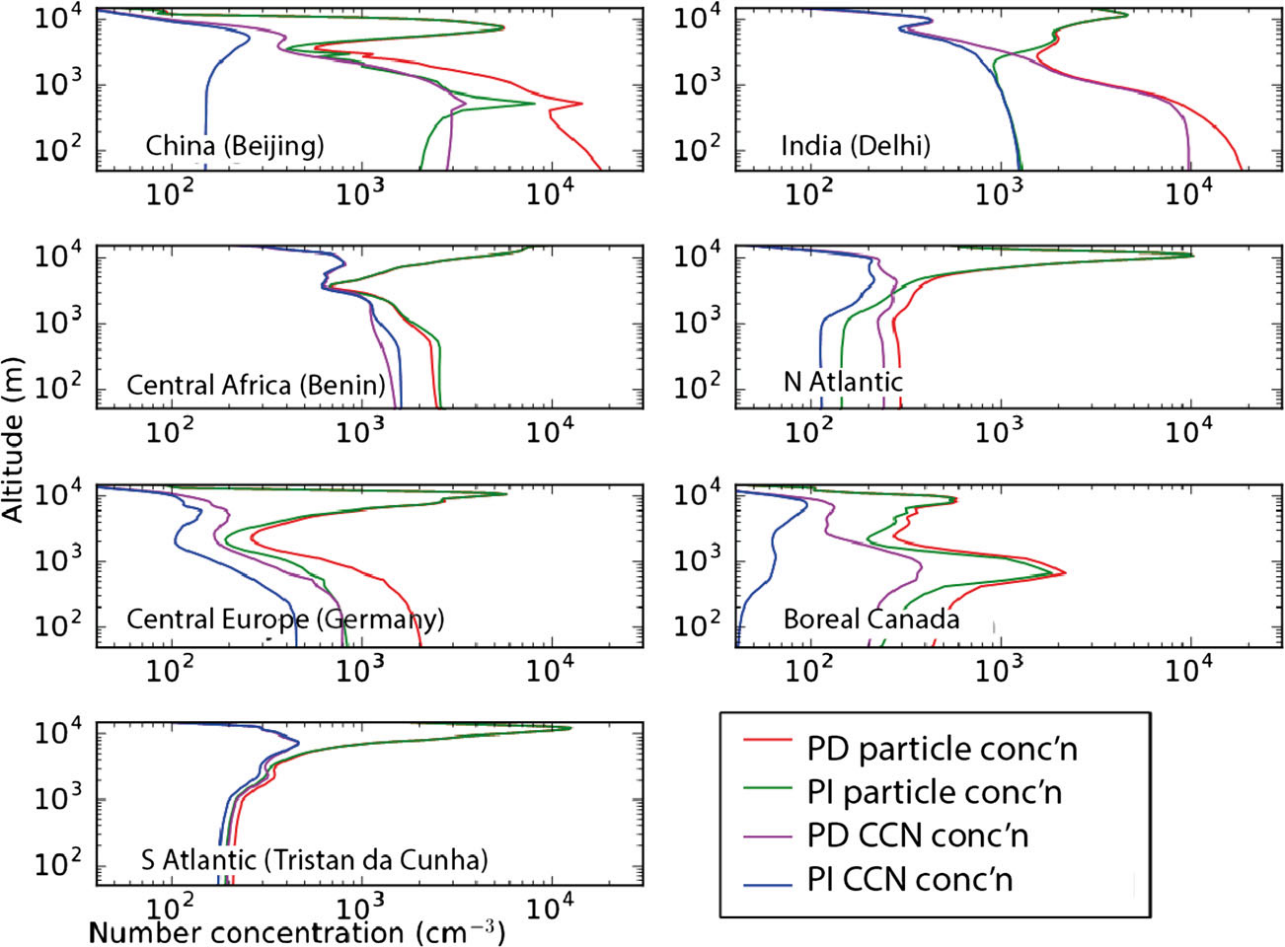
Model calculations of pre-industrial and present-day area-averaged aerosol vertical profiles over China, India, central African biomass-burning area, the North Atlantic, central Europe, boreal Canada, and the South Atlantic. Annual means of the total particle concentration (particles larger than 3 nm diameter) and CCN. Concentrations are for ambient temperature and pressure

Figure 5 shows the vertical profile of total particle number concentration (all particles greater than 3 nm diameter) and of CCN concentrations for a few locations around the world. The striking feature of these profiles is that PI and present-day concentrations are very similar above about 4 km altitude. At these altitudes, almost all aerosols originate from nucleation [92], which, in these simulations, is a binary sulphuric acid-water mechanism [113] that produces present-day particle concentrations in good agreement with observations. The insensitivity of free tropospheric aerosols to changes in emissions since the PI is expected based on what we know about the buffering of CCN concentrations in nucleation-dominated environments, although the effects of other species like organic compounds and ammonia [93, 97] are yet to be determined.

**Fig. 6:**
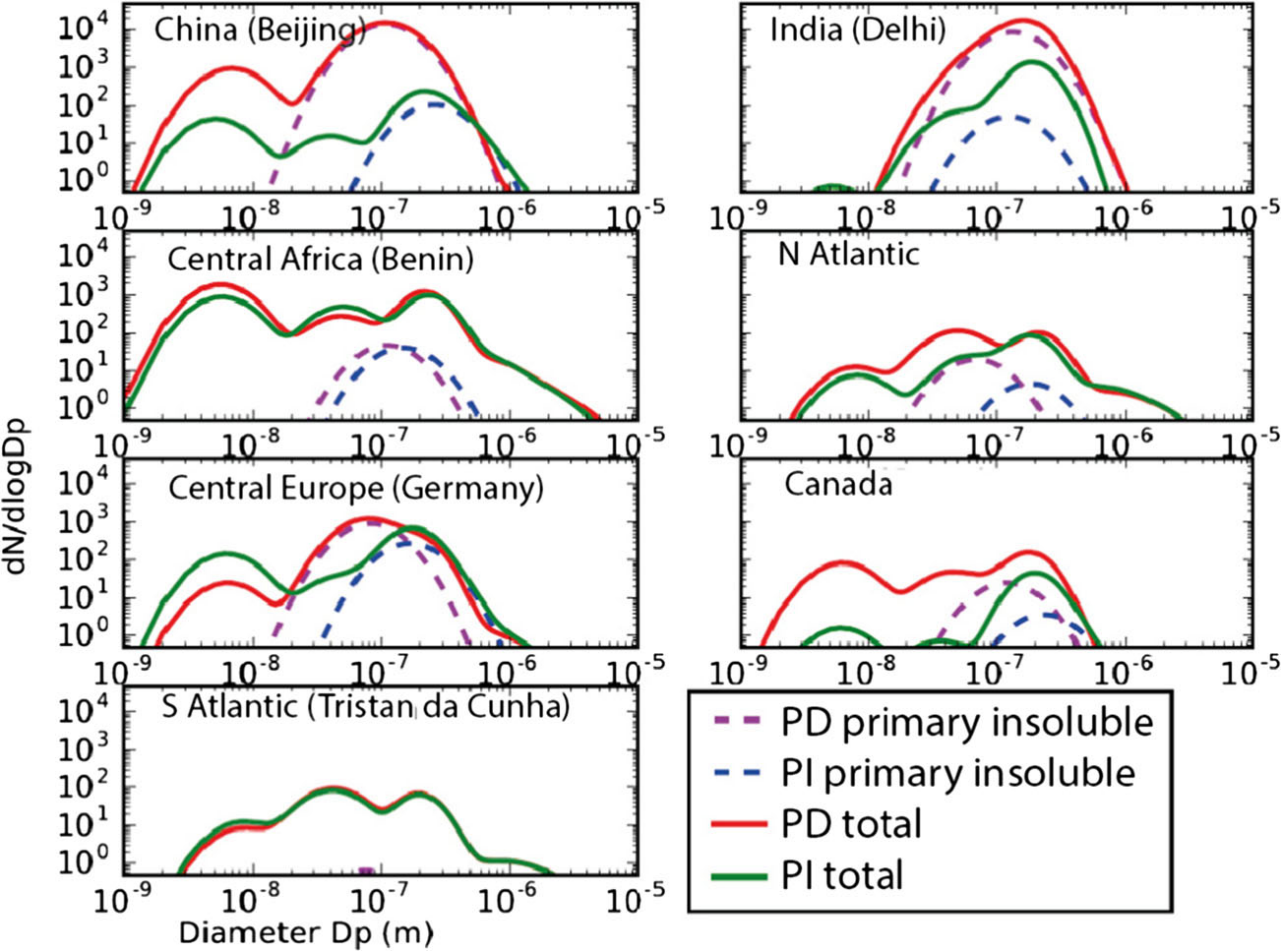
Model calculations of pre-industrial area-averaged annual mean aerosol size distribution for China, India, central African biomass-burning area, the North Atlantic, central Europe, boreal Canada, and the South Atlantic

Size distributions of aerosol change substantially between present-day and the PI (Fig. 6). Natural fire emissions are assumed to have a larger mode diameter than fossil fuel emissions, so there is a tendency towards smaller and more numerous particles in the present day than the PI. In Germany, this explains the downward shift in the diameter of the largest mode, but the nucleation mode is higher in the pre-industrial atmosphere, presumably due to the lower condensation sink. This is not true elsewhere, suggesting that the reduced condensation sink in the PI is accompanied by a reduction in condensable vapour concentrations.

**Fig. 7:**
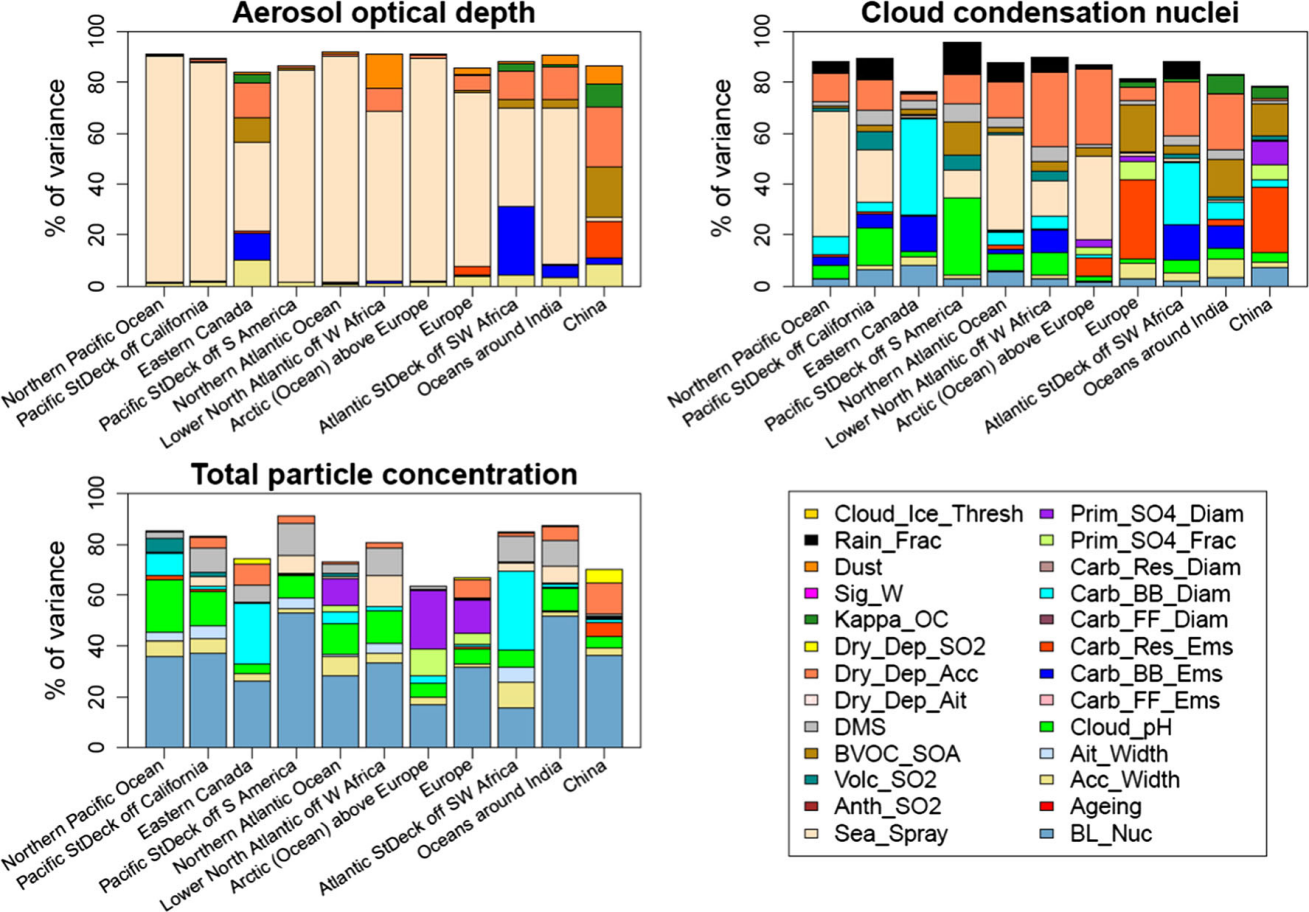
Causes of uncertainty in pre-industrial annual mean aerosol optical depth, CCN number concentration, and total particle number concentrations for the regions shown in Fig. 4. The *colours* from top to bottom follow the same order in the key. Where the fraction of variance is less than 100%, the remainder is caused by interactions between parameters

Figure 7 shows how different model processes and emissions contribute to the uncertainty in pre-industrial aerosol optical depth (AOD), CCN, and total number concentration in the 11 regions in Fig. 4. This analysis provides some indication of where improved knowledge will help to reduce model uncertainty. Causes of uncertainty vary spatially, as we have previously seen for present-day properties [111]. For AOD, the sea-spray emissions are the largest source of uncertainty in nearly all regions and completely dominate the uncertainty for oceanic regions. For the land-based regions such as eastern Canada and Europe, the biomass-burning emissions, the assumed accumulation mode width, the biogenic secondary aerosol formation from volatile organic compounds (BVOC_SOA), and the accumulation mode dry deposition velocity also make significant contributions to the pre-industrial AOD uncertainty. Dust emissions make a relatively small contribution to AOD uncertainty (less than 10–15% of total uncertainty) in the regions we have examined.

For CCN, the contributions to uncertainty are much more spatially varied, with many parameters having a large effect in at least one region. Over many oceanic regions, the sea spray aerosol emissions are the largest contributor, and for land-based regions, it is the carbonaceous residential emissions, the carbonaceous biomass-burning emissions (and the assumed particle diameter) that have the largest effect. We also see that the accumulation mode dry deposition velocity scale factor is a large contributor to the uncertainty across all regions.

For the total particle number concentration, the main contributions to uncertainty are reasonably similar across the regions. The boundary layer nucleation rate coefficient is the most important factor in nearly all regions. Also, the pH of cloud droplets, the mode diameter of new sub-grid sulphate particles, the diameter of emitted biomass-burning particles, and the dimethyl sulphide emissions each show large contributions within individual regions.

The main contributors to uncertainty in the PI are not the same as those for present-day conditions (plots not shown, but earlier studies are not greatly different [111]). The implication is that efforts to reduce uncertainty in present-day aerosol models will not directly translate into a reduced uncertainty in models of PI aerosols.

Our understanding of aerosol emissions and processes is still changing rapidly. In order to improve models of PI aerosols, we need to improve our understanding of processes that we already know to be uncertain, as outlined above, but also identify new aerosol processes or emissions that may be deficient or incomplete. A good example of such a new process, not reflected in the model simulations above, is pure biogenic nucleation [100], which could increase PI CCN concentrations by 4–19% [101]. These changes are comparable to the standard deviations in CCN concentrations caused by 26 other processes and emissions so would constitute a significant change to the model. Other nucleation mechanisms, like those involving iodine oxides [114–116], are also likely to have a larger relative effect on CCN in the PI atmosphere than today. A second example is the likelihood that emissions from fires in the PI were much higher than previously assumed [68]. Again, this structural change to the model would increase CCN and black carbon concentrations by over a factor of two over some northern hemisphere regions. The net effect of such missing processes is yet to be explored, but based on these studies, we can expect it to have a substantial effect on calculated radiative forcings.

## Open Questions and Future Research

The community needs to settle on a reliable and useful definition of “pre-industrial” aerosols. As we have described in this review, human activity was already perturbing the land and atmosphere from before the 1700s, and by 1850, early industrial emissions had already significantly affected aerosols in parts of the northern hemisphere, especially around the Atlantic. Many of the important factors affecting aerosols, as summarised in this review, are not considered in current climate model simulations, so it is not clear whether current definitions of pre-industrial are appropriate for aerosols [27]. Certainly, 1850 should be considered as “early industrial”, and the pre-industrial to present-day aerosol forcing is slightly lower when 1850 is used compared to using 1750 [6]. Although early anthropogenic emissions were low, the PI aerosol system was very susceptible to small changes [6]. We need to understand better how early changes in land use and land cover affected emissions and how much the aerosol system was perturbed by early industrial emissions. The substantial spatial variability in these changes could be very important depending on how the emissions ultimately affect sensitive CCN concentrations in cloudy regions.

Natural aerosol emissions are a major source of uncertainty in PI aerosols [6], and we cannot assume they were the same as today because of natural variability and the effects of human activity on land use and natural processes. To improve models, we need to develop a fundamental understanding of natural Earth system processes that control the key emissions such as dimethyl sulphide, marine organic emissions, and fires. Earth system model developments are needed to explore the biogeochemical cycles involving aerosols [78] as well as the biosphere’s response to climate change, which will have altered the emissions [2].

It is well understood that solar and volcanic radiative forcings were a major factor controlling PI climate variability [17]. However, variations in natural aerosols in the troposphere (such as through changes in fire emissions) should also be considered as a potential driver of climate variability in the PI because the aerosol-cloud system was more susceptible to small perturbations in emissions than it is today. To address this problem, we need to develop a much better understanding of variability in natural systems by building robust Earth system models.

There is a clear need to more fully explore the range of aerosol properties across multiple models because this has a direct bearing on the spread of multi-model ensembles in the Coupled Model Intercomparison Project (CMIP) that feeds into IPCC assessments. As shown by Wilcox et al. [7], there is scant information available on PI aerosols from CMIP5. The AerchemMIP project [117] provides an opportunity for such an analysis. It will also be important to establish the extent to which PI aerosol properties directly affect modelled aerosol-cloud forcings, or whether artificial restrictions on cloud drop concentrations [20] remove some of the sensitivity.

The lack of direct measurements of aerosol properties with global coverage and under different meteorological conditions makes models very reliant on a sound understanding of aerosol physical and chemical processes, which were probably different in the PI compared to today. Developments in all of these model components are needed. As shown by Gordon et al. [101], progress is being made in exploiting well-designed chamber measurements to understand the mechanism of particle nucleation under PI conditions. However, a much deeper knowledge of PI gas phase and aerosol chemistry is needed to refine our understanding of these and other aerosol processes.

There is scope to make more use of ice core records to evaluate Earth system models and extract new information about PI aerosol chemistry and distribution. This analysis is necessary because records suggest natural PI aerosols were not the same as today. We need more model analyses of existing data as well as novel ways of extracting more aerosol information from cores. A particular challenge will be to relate point measurements to regional aerosol emissions and processes [40].

The change in ice-nucleating particle concentrations and the effect on cloud glaciation and planetary radiative balance remain essentially completely open. Progress is being made by including species-specific ice-nucleating particles in global models [118–120] so that they can be simulated based on changing aerosol emissions. However, our overall understanding of ice-nucleating particles is still evolving [22, 23], and it is not known how the specific ice-active components may have been different in the PI.

In addition to understanding the properties of aerosols in the PI, we also need to understand how they interacted with clouds to affect cloud physical properties, precipitation, and planetary energy balance. In particular, we need to understand whether low aerosol concentrations in the PI affected aerosol removal and hence fed back on the aerosol number concentrations. These processes are currently poorly handled in most global models. Cloudy regions with aerosol number concentrations occasionally close to PI conditions can be found in today’s atmosphere [38] and could be studied as analogues.

Finally, as our understanding of PI aerosols improves, we need to assess their effects on atmospheric dynamics and climate sensitivity. It is known that monsoons and the position of the ITCZ respond to hemispheric-scale forcing from volcanic and anthropogenic aerosols [121, 122]. Changes in PI aerosols against a baseline of fairly low concentrations could have substantial regional radiative effects. These may affect the distribution of ocean heat during the spin up of climate models and thereby affect the whole historical simulation [39] and the model’s climate sensitivity [8–10].

## Conclusions

It is clear that progress is being made on understanding the state and behaviour of aerosols in the PI. However, at present, parts of the problem are being studied in isolation (e.g. nucleation [101]). Given the complex interactions in the aerosol system, the interactions of aerosols with clouds [18], and with the wider Earth system [2, 78], it is essential to build and evaluate more complete models with a focus on the PI. The current generation of Earth system models provides a good basis for such research. As we develop these models further, we need to be aware that the climate may be very sensitive to model errors [6], neglected processes [101], and emissions [68]. Improvement in the realism of the models may also require the inclusion of some complex physical and chemical processes, which will be difficult to verify against present-day measurements [101].

The study of PI aerosols has been a relatively neglected area of climate science, with most effort being directed to understanding historical changes since the PI era [104, 123] even though the starting point for these simulations has not been well defined [6, 7]. The development of more sophisticated models provides an opportunity to understand the PI aerosol system better, although the evaluation of the models will be very challenging.
